# Pharmacokinetic Comparison between Methotrexate-Loaded Nanoparticles and Nanoemulsions as Hard- and Soft-Type Nanoformulations: A Population Pharmacokinetic Modeling Approach

**DOI:** 10.3390/pharmaceutics13071050

**Published:** 2021-07-09

**Authors:** Seung-Hyun Jeong, Ji-Hun Jang, Yong-Bok Lee

**Affiliations:** College of Pharmacy, Chonnam National University, Gwangju 61186, Korea; rhdqn95@naver.com (S.-H.J.); jangji0121@naver.com (J.-H.J.)

**Keywords:** methotrexate-loaded nanoparticles, methotrexate-loaded nanoemulsions, hard-type nanoformulation, soft-type nanoformulation, population pharmacokinetic modeling

## Abstract

The purpose of this study was to identify and explore the differences in pharmacokinetics between different nanoformulations. This was done by comparing the pharmacokinetics of methotrexate-loaded nanoparticles [poly(lactic-co-glycolic acid); size of 163.70 ± 10.25 nm] and nanoemulsions (olive oil and Labrasol; size of 173.77 ± 5.76 nm), which represent hard- and soft-type nanoformulations, respectively. In addition, the population pharmacokinetic modeling approach as a useful tool for the comparison of pharmacokinetics between nanoformulations was newly proposed through this study. Significant pharmacokinetic differences were identified between nanoformulations through the new population pharmacokinetic modeling approach. As a result, the formulation type was explored as a significant covariate. The clearance and bioavailability of methotrexate-loaded nanoemulsions tended to decrease by 99% and increase by 19%, respectively, compared to those of the nanoparticles. The exploration of significant pharmacokinetic differences between drug formulations and their correlations presented in this study provide new perspectives on the development of nanoformulations.

## 1. Introduction

The pharmacokinetic properties of general small-molecule chemicals and those enclosed in nanoformulations are very different. These pharmacokinetic differences may occur because the distribution characteristics of nanoformulations dominate those of small-molecule chemicals in the body. In particular, the characteristics of nanoformulations are remarkable when the compounds are administered intravenously. In the case of orally administered nanoformulations, it is common to release small-molecule chemicals from nanoformulations so that these chemicals are absorbed in the gastrointestinal tract. However, a mechanism by which the nanoformulation itself is absorbed (through Peyer’s patch M-cells) also exists in the gastrointestinal tract [1,2]. The possibility that some nanoformulations that are not completely degraded are transported within the cell when passing through the M-cells has also been raised. For example, it has been qualitatively and quantitatively confirmed that topotecan-loaded nanoparticles are transferred to the lymphatic tissues when administered orally [3].

Research into changes in the pharmacokinetic properties of nano-sized formulations of various drugs is actively being conducted [4,5,6,7,8,9,10,11,12,13,14,15,16]. Most nanoformulation studies have reported that the bioavailability of nanoformulated drugs is largely improved and delayed-release patterns are observed in comparison to the standard drugs used as controls [4,5,6,7,8,9,10,11,12,13,14,15,16]. These results clearly imply that changes of in vivo pharmacokinetics are associated with the nanoformulation of the drug. Most nanoformulation studies reported to date have made major comparisons between standard formulations (such as solutions) and nanoformulations [4,5,6,7,8,9,10,11,12,13,14,15,16]. Those studies reported major improvements in the pharmacokinetic properties of the nanoformulations. However, a simple comparison of the pharmacokinetic parameters and bioavailability obtained by administering conventional small-molecule chemicals and nanoformulations only demonstrates the relative pharmacokinetic improvement of the nanoformulations compared to the conventional chemicals. Therefore, it would be desirable to check the pharmacokinetic properties of nanoformulation-encapsulated drugs, and then compare the differences in pharmacokinetics between the nanoformulations. This would allow us to break away from the existing framework and explore new pharmacokinetic properties through comparisons between different nanoformulations.

There have been few reports of pharmacokinetic comparisons between nanoformulations. Even in comparisons between standard formulations and nanoformulations, only the statistically significant differences in the pharmacokinetic parameters through non-compartmental analysis have been compared. However, in the current situation where various nanoformulations are actively under investigation, the ability to compare the properties of these various nanoformulations and the presentation of an effective tool are thought to be very useful reference techniques for the development of future nanoformulations. Therefore, in this study, we attempted to analyze pharmacokinetic differences between nanoformulations through generally used non-compartmental model approach and newly applied population pharmacokinetic model approach. A comparative study between nanoformulations, which can be a useful reference for strategic selection of nanoformulations with the better pharmacokinetic properties, is considered very necessary and urgent.

The main purpose of this study was to divide methotrexate-loaded nanoformulations into nanoemulsions and polymeric nanoparticles, i.e., soft- and hard-type formulations, and to compare them. According to a previous report [17], most nanoformulations can be classified as either hard- or soft-type nanoformulations according to their physical traits, such as the external flexibility of the nanoformulation. The authors suggested that studies on the difference in in vivo pharmacokinetics between these two types should be carried out in the future [17]. A difference in the external flexibility of a nanoformulation is thought to lead to effective pharmacokinetic differences (as the nanoformulation would behave differently in the body). In this paper, to enable a comparison between nanoformulations, the pharmacokinetics of nanoformulations were identified through a population pharmacokinetic approach, and population pharmacokinetic model analysis including covariates was performed. Through a comparison between soft- and hard-type nanoformulations and exploration of effective covariates, we suggest which formulation would be more efficient in terms of pharmacokinetics, and attempt to aid in the development of new nanoformulations in the future. For this study, based on the results of administration of methotrexate-loaded nanoparticles [6] and nanoemulsions [7] in rats, the pharmacokinetic difference between these nanoformulations and methotrexate solution was first investigated through population pharmacokinetic analysis. Next, we identified the pharmacokinetic differences between nanoparticles (hard-type) and nanoemulsions (soft-type) containing methotrexate using a population pharmacokinetic modeling approach. 

Using the pharmacokinetic parameters calculated by non-compartmental analysis, it is possible to simply compare which parameter values are larger or smaller depending on the formulation groups. However, the population pharmacokinetic modeling approach has the advantage of more objectively and clearly comparing the pharmacokinetic differences between formulations, as it takes into account the interindividual variability (IIV) and intra-individual errors in each formulation administration group. In particular, when the pharmacokinetic difference between nanoformulations is difficult to clearly judge by a simple pharmacokinetic profile-based comparison or using the parameters from non-compartmental analysis, a population pharmacokinetic modeling approach (using nonlinear mixed effects) that takes into account the overall factors and differences in variability will be very useful. In addition, through a population pharmacokinetic modeling approach, it is possible to objectively identify and explore the correlations between parameters for specific covariates (such as the formulation type), which are limited to non-compartmental analysis. Given this information, it is possible to predict the pharmacokinetics of a molecule according to the type of formulation, and provide a basis for judgment that will be useful for the selection of nanoformulations in the future. This comparison of the pharmacokinetics of hard- and soft-type nanoformulations has not previously been performed. This research is expected to provide a useful reference for further development of nanoformulations with significantly improved pharmacokinetic properties and to improve our understanding of the physical properties of nanoformulations.

## 2. Methods

### 2.1. Data Collection

Previous formulation studies have aimed to identify and compare differences in pharmacokinetics between free methotrexate solution and methotrexate-loaded nanoformulation administration groups [6,7]. These were pharmacokinetic studies on methotrexate-loaded nanoparticles [poly(lactic-co-glycolic acid)] and nanoemulsion formulations (olive oil and Labrasol), as well as free methotrexate solution as a control. Therefore, we attempted to compare the pharmacokinetic differences between methotrexate-loaded nanoparticles and nanoemulsions (and free methotrexate solution) using a population pharmacokinetic analysis method. The total number of individual rats used in the analysis was 40. Male Sprague-Dawley rats weighing 240–260 g were used, all of which were 7–9 weeks old. All rats in the formulation studies were physically healthy individuals without disease. All animal experiments were thoroughly reviewed and approved by the Chonnam National University Animal Experimental Ethics Committee, Gwangju, Republic of Korea. The permit number for the animal experiments was CNU IACUC-YB-2017-47. All procedures were conducted according to the revised Guidelines for Ethical Conduct in the Care and Use of Animals and the rules of Good Laboratory Practice.

### 2.2. Pharmacokinetic Analysis

The base pharmacokinetic parameters for the groups administered free methotrexate solution and methotrexate-loaded nanoformulations were calculated by non-compartmental analysis using Phoenix WinNonlin (8.3 version, Pharsight, Certara Inc., Princeton, NJ, USA) software. The area under the curve from 0 h to infinity (AUC_0–∞_) was calculated as the sum of AUC_0–t_ and C_last_/k, where C_last_ is the last measurable concentration, t is the time of C_last_, and k is the elimination rate constant at terminal phase. AUC_0–t_ was calculated using a linear trapezoidal rule from 0 to t h after oral or intravenous administration. The half-life (T_1/2_) was calculated as 0.693/k, and the volume of the distribution (V) was calculated as dose/k • AUC_0–∞_. The clearance (CL) was calculated by dividing the intravenous dose (as 5 or 0.024 mg/kg) of methotrexate by AUC_0–∞_. The mean residence time (MRT) was calculated by dividing the area under the first moment curve (AUMC) of methotrexate by AUC_0–∞_. Here, AUMC indicates the product of concentration and time integrated over time. When a steady state was reached, the predicted volume of distribution (V_ss_) was calculated as MRT • CL. The quantitative results of methotrexate obtained using ultra-high-performance liquid chromatography-electrospray ionization-mass spectrometry (UHPLC-ESI-MS/MS) were plotted as graphs of plasma concentration (*y*-axis) of methotrexate over time (*x*-axis). Additionally, the highest drug concentration in plasma (C_max_) and the time to reach C_max_ (T_max_) were determined from the plasma methotrexate concentration-time curve of each individual. All pharmacokinetic parameter values were estimated as mean ± standard deviation (SD).

### 2.3. Population Pharmacokinetic Model Development

Construction and analysis of the population pharmacokinetic model of methotrexate-loaded nanoparticles and nanoemulsions (and free methotrexate solution) were performed using a nonlinear mixed effects (NLME) model approach using Phoenix NLME (version 8.3, Pharsight, Certara Inc., Princeton, NJ, USA) software. This is a popular choice for population pharmacokinetic analysis of drugs [18,19]. Population pharmacokinetic model development was performed using the first-order conditional estimates method with extended least squares estimation (with *ŋ*–*ε* interaction). Three steps were used to construct and analyze the population pharmacokinetic model. The first step was to establish a base model that could explain the pharmacokinetics of free methotrexate solution and methotrexate-loaded nanoformulations. The second step was to explore the effectiveness of the formulation as a significant candidate covariate that could account for the pharmacokinetic variability between administration groups. Lastly, the third step was to establish a population pharmacokinetic model that could adequately explain the experimentally obtained pharmacokinetic results by applying the explored covariate (difference in formulation) to the base model established in the previous step. To establish a compartment model suitable as a base model structure, the data on methotrexate concentration in plasma over time in each individual were applied to various compartment models. One-, two-, and three-compartment disposition models were tested. Zero- or first-order absorption/elimination was attempted regarding the transit rate of drug between compartments. Regarding oral absorption of free methotrexate solution and methotrexate-loaded nanoformulations, models with or without absorption lag-time and multiple transit models were tried. The final selection of structural base model was based on the statistical differences between models using Akaike’s information criterion (AIC), goodness-of-fit plots, and twice the negative log likelihood (−2LL). The change in statistical significance according to the increase or decrease in the number of parameters used in the model was also considered. Additive, log-additive, proportional, mixed, and power error models were applied to explain residual variability. The IIV in pharmacokinetic parameters was evaluated using an exponential error model, as shown in the following equation: P_i_ = P_tv_ • exp(*ŋ*_i_), where *ŋ*_i_ is the random variable for the ith individual, which was normally distributed with mean 0 and variance *ω*^2^; P_i_ is the parameter value of the ith individual, and P_tv_ is the typical value of the population parameter. We confirmed that considering IIV in each parameter markedly improved the model. Formulation differences were applied as a potential covariate to account for the pharmacokinetic variability of methotrexate between administration groups, i.e., the pharmacokinetic parameters calculated by non-compartmental analysis for each administration group were primarily screened to assess the degree of correlation between formulations and parameters through statistical analysis. The statistical tool used here was Student’s *t*-test. A *p* value of <0.05 confirmed by Student’s *t*-test test was taken to indicate a significant difference. Subsequently, the formulation differences were sequentially applied to the IIV model as the selected candidate covariate. Then, the effect of the covariate was assessed using exponential, additive, and power options. The covariates were included or eliminated by stepwise backward elimination and forward addition. The inclusion of covariates was determined by the change in the objective function value (OFV). Covariates corresponding to a decrease in the OFV value of greater than 3.84 (*p* < 0.05) were included in the base model (in the forward addition procedure). In addition, covariates corresponding to a decrease in OFV of greater than 6.63 (*p* < 0.01) through the backward elimination process were not removed from the model and were included.

### 2.4. Population Pharmacokinetic Model Evaluation

The final established population pharmacokinetic model of methotrexate-loaded nanoparticles and nanoemulsions (and free methotrexate solution) was evaluated and validated both visually and numerically. All model evaluation and validation processes were performed using Phoenix NLME and R software (R Core Team). The evaluation of the model was largely done using the following four methods: goodness-of-fit (including distribution of residuals), visual predictive check, bootstrapping, and normalized prediction distribution error. The goodness-of-fit was confirmed using diagnostic scatter plots, as follows: (A) population-predicted concentrations (PRED) versus observed (DV), (B) individual-predicted concentrations (IPRED) versus DV, (C) PRED versus conditional weighted residuals (CWRES), (D) time (IVAR) versus CWRES, and (E) quantile–quantile plot of the components of CWRES. Visual predictive check of the final established model was performed using the visual predictive check option of Phoenix NLME. The number of simulations for the visual predictive check was 1000. The IVAR–DV concentration data were graphically superimposed on the median values and the 5th and 95th percentiles of the IVAR-simulated concentration profiles. If the DV concentration data were approximately distributed within the 95th and 5th prediction interval, the model was declared to be precise. The stability of the final model was confirmed using non-parametric bootstrap analysis with the bootstrap option of Phoenix NLME. A total of 1000 replicates were generated by repeated random sampling with replacement from the original dataset. The estimated parameter values, such as the standard errors (SEs, including confidence intervals) and medians from the bootstrap procedure, were compared with those estimated from the original dataset. Normalized prediction distribution error was used to evaluate the predictive performance of the model based on a Monte Carlo simulation with the R package. The normalized prediction distribution error results were summarized graphically using (A) a quantile–quantile plot of the normalized prediction distribution error, (B) a histogram of the normalized prediction distribution error, (C) a scatterplot of normalized prediction distribution error versus IVAR, and (D) a scatterplot of normalized prediction distribution error versus PRED. If the predictive performance is satisfied, the normalized prediction distribution error will follow a normal distribution (Shapiro–Wilk test) with a mean value of zero (*t*-test) and a variance of one (Fisher’s test).

## 3. Results and Discussion

### 3.1. Comparison of Pharmacokinetic Results between Free Methotrexate Solution and Methotrexate-Loaded Nanoformulations

**Figure 1:**
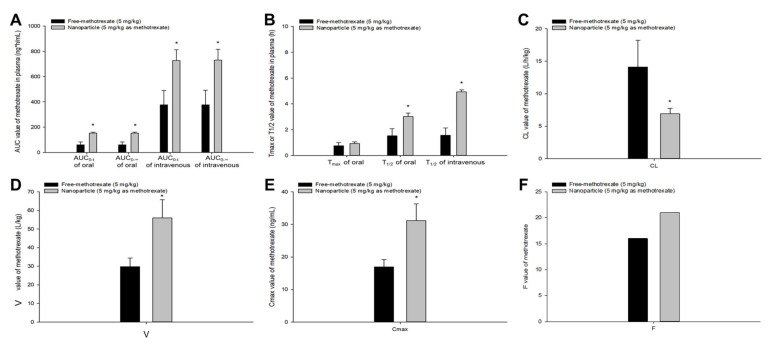
Mean pharmacokinetic parameters (**A**), AUC_0-t_ and AUC_0-∞_; (**B**), T_max_ and T_1/2_; (**C**), CL; (**D**), V; (**E**), C_max_; (**F**), F of methotrexate after oral or intravenous administration of free methotrexate solution (black box, 5 mg/kg) and methotrexate-loaded nanoparticles (gray box, 5 mg/kg as methotrexate) in rats. Vertical bars represent standard deviation of the mean (*n* = 5). * *p* < 0.05 compared with the administration of free-methotrexate.

**Figure 2:**
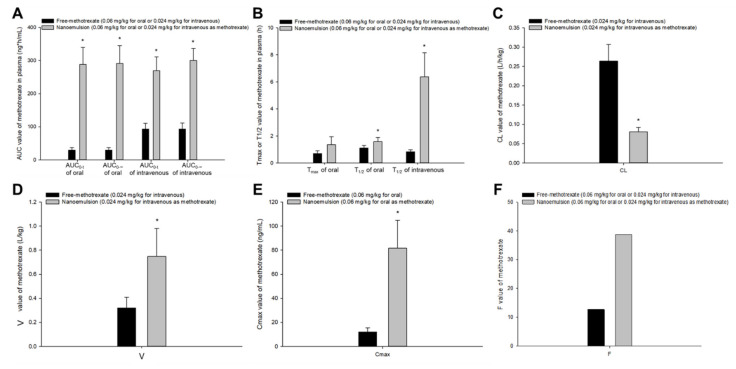
Mean pharmacokinetic parameters (**A**), AUC_0-t_ and AUC_0-∞_; (**B**), T_max_ and T_1/2_; (**C**), CL; (**D**), V; (**E**), C_max_; (**F**), F of methotrexate after oral or intravenous administration of free methotrexate solution (black box, 5 mg/kg) and methotrexate-loaded nanoemulsions (gray box, 0.06 or 0.024 mg/kg as methotrexate) in rats. Vertical bars represent standard deviation of the mean (*n* = 5). * *p* < 0.05 compared with the administration of free-methotrexate.

The comparison of in vivo pharmacokinetic profiles and parameters (non-compartmental analysis) revealed significant differences between the free methotrexate solution and methotrexate-loaded nanoformulations; this confirmed the results of previous studies [6,7]. Figure 1 shows a graphical comparison of pharmacokinetic parameter values according to oral or intravenous administration of free methotrexate solution and methotrexate-loaded nanoparticles. The calculated parameter values of AUC, T_1/2_, V, and C_max_ were significantly higher in methotrexate-loaded nanoparticles than free methotrexate, whereas CL values were significantly lower in nanoparticles. These pharmacokinetic results suggest that use of methotrexate-loaded nanoparticles could result in significant improvements in blood pharmacokinetic profile and bioavailability compared to use of free drug. Figure 2 shows a graphical comparison of pharmacokinetic parameter values according to oral or intravenous administration of free methotrexate solution and methotrexate-loaded nanoemulsions. The parameters, AUC, T_1/2_, V, and C_max_, were significantly higher in nanoemulsions than in free methotrexate. On the other hand, the calculated CL value was significantly lower in methotrexate-loaded nanoemulsions than free drug. Compared to the free methotrexate, both nanoparticles and nanoemulsions resulted in higher mean T_max_ values of methotrexate (although the differences were not significant). However, methotrexate-loaded nanoparticles and nanoemulsions differed in the degree of increase or decrease in the pharmacokinetic parameters compared to free drug. The overall changes in AUC, T_max_, T_1/2_, V, CL, and C_max_ compared to free drug were about 1.5–5 times greater when methotrexate-loaded nanoemulsions were used rather than methotrexate-loaded nanoparticles. Even in terms of the F value, nanoparticles and nanoemulsions showed a difference of 1.31- and 3.05-fold, respectively, compared to free methotrexate. These pharmacokinetic comparison results suggest that there may be significant pharmacokinetic differences between methotrexate-loaded nanoparticles and nanoemulsions.

### 3.2. Population Pharmacokinetic Modeling Approach to Comparing Free Methotrexate Solution and Methotrexate-Loaded Nanoformulations

A population pharmacokinetic modeling approach using NLME was applied to determine whether there is a pharmacokinetic difference between methotrexate-loaded nanoformulations (including nanoparticles and nanoemulsions) and free methotrexate solution (as a control), if so, to what extent, and what specific pharmacokinetic differences occur. All plasma concentration data were simultaneously fitted to the same model. The type of formulation (nanoformulation or not) was set as a covariate of the model, and this covariate was found to be effective in the population pharmacokinetic model of methotrexate-loaded nanoformulations and free methotrexate solution. When applying the plasma concentration-time data of nanoformulations and free methotrexate solution (according to oral or intravenous administration) to the base compartment models for each, 1-compartment model with absorption lag-time and first-order absorption/elimination was the most suitable. In addition, the oral bioavailability (F) and the absorption rate constant (K_a_) were reflected in the base structural model. The CL and V in the model represent the drug elimination and volume of distribution from the compartment, respectively. As a result, the pharmacokinetic parameters used in the base structural model were as follows: clearance for the central compartment (CL), volume of distribution for the central compartment (V), first oral absorption rate constant (K_a_), absorption lag-time (T_lag_), and oral bioavailability (F). Though we attempted to apply a two- or three-compartment model, these models could not be applied to many subjects. However, the 1-compartment model was applicable to all experimental groups. The intra-subject variability was best explained using a log-additive error model. For the parameters CL, V, K_a_, T_lag_, and F in the base model, IIV was sequentially considered or excluded to determine whether the exponential error IIV model application is appropriate. As a result, the model that considered IIV for all those parameters (CL, V, K_a_, T_lag_, and F) was the most suitable. Although the total number of estimated parameters decreased when IIV was excluded for each of the parameters (CL, V, K_a_, T_lag_, and F), the AIC and -2LL values increased significantly without resulting in significant model improvement. Table 1 summarizes the steps that were taken to develop the base structural model for the groups administered free methotrexate solution and methotrexate-loaded nanoformulations. The effect of covariates on the pharmacokinetic parameters of free methotrexate solution and methotrexate-loaded nanoformulations was evaluated by sequentially applying the covariates to the base structural model established in the previous step. The covariate here was whether the drug took the form of a nanoformulation, as mentioned earlier. This covariate was reflected as categorical data. The fit of the model was judged by the significance of the change in OFV values compared to the base models of free methotrexate solution and methotrexate-loaded nanoformulations (without covariates). When the nanoformulation in T_lag_ was considered to be a covariate, the OFV value increased with the increase in the number of parameters to be estimated. This implies that there was no significant difference in T_lag_ for free methotrexate solution and methotrexate-loaded nanoformulations when using a model-based approach. The inclusion of nanoformulation in the base model as a covariate for each of the parameters V, CL, K_a_, and F (forward addition) resulted in an OFV that was lower than the significance criterion of 3.84 (*p* < 0.05). In particular, the decrease in OFV was greatest when nanoformulation was considered to be a covariate of CL. This reduction produced a value that was lower than 6.63 (*p* < 0.01), which was the criterion for model fit by backward elimination. Therefore, these results imply that the use of nanoformulation as a covariate for CL is appropriate and valid. In addition to CL, the model fit by forward addition and backward elimination was lower than the reference values (3.84 or 6.63), despite an increase in the number of parameters that needed to be estimated, when applying nanoformulation as a covariate of V, F, and K_a_. Therefore, nanoformulation was reflected in the parameters of V, CL, K_a_, and F as an effective covariate of the final population pharmacokinetic model for free methotrexate solution and methotrexate-loaded nanoformulations. Table 2 summarizes the covariate selection process for the final population pharmacokinetic model of free methotrexate solution and methotrexate-loaded nanoformulations according to OFV. The equation of the final population pharmacokinetic model was as follows:

V = tvV · (1 + dVdFormulation · formulation type _Free methotrexate = 0; Nanoformulation = 1_) · exp(*ŋ*_V_) (1)

CL = tvCL · (1 + dCLdFormulation · formulation type _Free methotrexate = 0; Nanoformulation = 1_) · exp(*ŋ*_CL_) (2)

T_lag_ = tvT_lag_ · exp(*ŋ*_Tlag_) (3)

K_a_ = tvK_a_ · (1 + dKadFormulation · formulation type _Free methotrexate = 0; Nanoformulation = 1_) · exp(*ŋ*_Ka_) (4)

F = tvF · (1 + dFdFormulation · formulation type _Free methotrexate = 0; Nanoformulation = 1_) · exp(*ŋ*_F_) (5)

Equations (1)–(5) mean the equations for V, CL, T_lag_, K_a_, and F in the final population pharmacokinetic model (for free methotrexate solution and methotrexate-loaded nanoformulations), respectively. In the case of free methotrexate, the value of formulation type is 0, and in the case of a nanoformulation (including nanoemulsions and nanoparticles), the value of formulation type is 1. 

Here, the formulation refers to the difference between a free drug and a nanoformulated drug containing both nanoparticles and nanoemulsions.

The parameter values estimated by the population pharmacokinetic model of free methotrexate solution and methotrexate-loaded nanoformulations are presented in Table 3. The relative standard error (RSE, %) values of all parameters estimated in the final population pharmacokinetic model were 0.014–45.227%. The Eta shrinkage (%) values for V, CL, T_lag_, K_a_, and F were 0.173–0.600%, which were acceptable. The IIV (%) of V, CL, K_a_, and F were relatively low at 0.252, 58.130, 0.002 and 62.161%, respectively; these numbers were lower than the IIV values of the previously established base model. The IIV values for V, CL, K_a_, and F in the previously established base model were 0.277, 97.658, 0.003, and 76.800%, respectively. This indirectly implied that in the final model of population pharmacokinetics of free methotrexate solution and methotrexate-loaded nanoformulations, it was appropriate to consider nanoformulation as a covariate for V, CL, K_a_, and F. The degrees of correlation between each of these parameters and nanoformulation were 0.429, −0.355, 10.883, and 4.246, respectively. This was similar to the results of the previous comparison of parameters between free methotrexate solution and methotrexate-loaded nanoformulations (Figure 1 and Figure 2), i.e., in the final model, the correlations between V, K_a_, and F and nanoformulation were positive, and the correlation between CL and nanoformulation was negative. This agreed with the results of the non-compartmental analysis, which showed that methotrexate-loaded nanoformulations had higher V, F, and C_max_ and lower CL values than free methotrexate solution. As a result, the covariate search results indicating that V and F increased, and CL decreased when the nanoformulations of methotrexate were used were reflected in the final population pharmacokinetic model. Population pharmacokinetic modeling confirmed that use of a nanoformulation of the drug induced significant pharmacokinetic changes and the formulation type (nanoformulated or not) was significantly correlated with V, F, CL, and K_a_. Although it was difficult to confirm the correlations between pharmacokinetic parameters and formulation type by either non-compartmental analysis or a simple comparison of pharmacokinetic profiles, these relationships could be confirmed through the population pharmacokinetic modeling approach. In addition, unlike non-compartmental analysis, where the ability to conduct comparisons according to the route of administration is limited, population pharmacokinetic modeling clearly identifies the differences in groups (considering interindividual and/or intra-individual variability) by a covariate such as the formulation type (nanoformulated or not), regardless of the route of administration. Although the T_lag_ estimated by the final model was close to 0, considering T_lag_ as a parameter in the base model was reasonable in terms of -2LL and AIC, as mentioned above. This indirectly implies that the T_lag_ estimated in the final model was not very important to the interpretation of the pharmacokinetic differences between formulations (although consideration of T_lag_ was suitable for the base model structural stability). The Eta values of V, T_lag_, and K_a_ estimated in the final model were close to 0. This implies that the amount of diversity among individuals for V, T_lag_, and K_a_ was not significantly considered during the process of parameter estimation in the final model, or that the amount of diversity among individuals in the corresponding parameters according to the model setting (with consideration of the covariate) was greatly reduced. In the current study, only the type of formulation was considered to be a covariate to explain the pharmacokinetic diversity (especially for CL and F), but it may be possible to effectively explain the interindividual diversity in V, T_lag_, and K_a_ through exploration of additional effective covariates (such as genetic factors related to absorption) in the future. In addition, correlations with other parameters were assessed through the OMEGA block for V, T_lag_, and K_a_, but no clear correlations (correlation coefficients < 0.1) were confirmed.

Here, the formulation refers to the difference between a free drug and a nanoformulated drug containing both nanoparticles and nanoemulsions.

**Figure 3:**
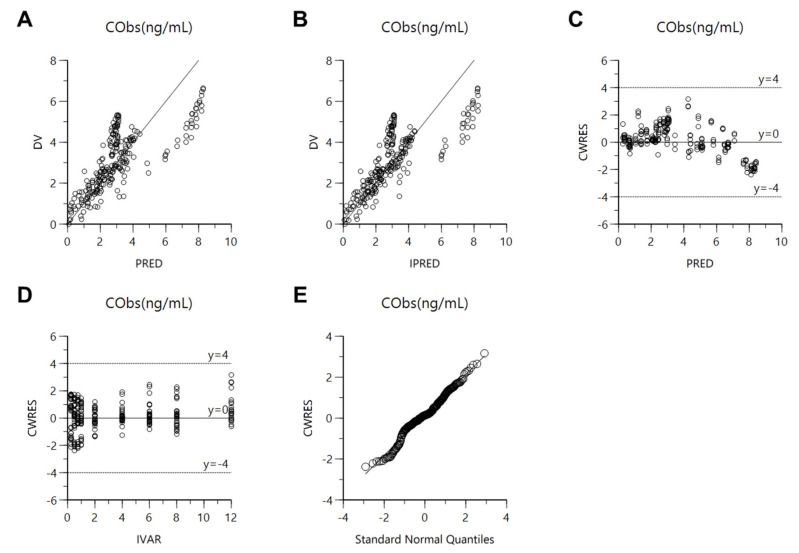
Goodness-of-fit plots of the final population pharmacokinetic model for methotrexate-loaded nanoformulation and free methotrexate solution. (**A**) Population-predicted concentrations (PRED) vs. observed plasma concentration (DV); (**B**) Individual-predicted concentrations (IPRED) vs. DV; (**C**) PRED vs. CWRES; (**D**) Time (IVAR) vs. CWRES; (**E**) Quantile–quantile plot of the components of CWRES.

Established population pharmacokinetic models of free methotrexate solution and methotrexate-loaded nanoformulations were roughly evaluated for goodness-of-fit plots and bootstrapping. Figure 3 shows the goodness-of-fit results for the final population pharmacokinetics model. The methotrexate concentration predicted by the population pharmacokinetic model, in the population or individual, showed relatively good agreement with the experimental observations. Nevertheless, the model’s estimates were larger than the experimental values in the high-concentration area, possibly because the current model has limitations in sufficiently explaining the data obtained after the administration of free methotrexate solution and nanoformulations, i.e., due to the considerable variability in plasma concentration values obtained after intravenous or oral administration of free methotrexate solution and nanoformulations, the diversity of dosages, and the limited covariate values, it was difficult to accurately estimate the parameter values and simultaneous fittings in the model. In the future, the model could be improved through the reflection of additional effective covariates (in addition to nanoformulation) that can explain the pharmacokinetic variability between the free methotrexate solution and nanoformulations administration groups. CWRES were well-distributed symmetrically with respect to zero, i.e., CWRES were well-distributed at random without any specific bias. Additionally, the CWRES’s values at all points of predicted concentration or time in the population did not deviate from ±4. The quantile–quantile plot of the components of CWRES was close to a straight line where the *x*- and *y*-axes were symmetrical. Consequently, the goodness-of-fit plot results presented in Figure 3 suggest that the final established population pharmacokinetic model of free methotrexate solution and methotrexate-loaded nanoformulations had no graphically significant problems. Nevertheless, the observed wave pattern is most likely related to the practical limitations of the current model, as mentioned above. Table 4 shows the bootstrapping results for the final established population pharmacokinetic model of free methotrexate solution and methotrexate-loaded nanoformulations. All the parameter values estimated in the final model were within the 95% confidence interval of the bootstrap analysis (number of replicates, 1000). Additionally, the estimated values of the model parameters were similar to the median estimated by the bootstrap analysis. As a result, bootstrapping analysis confirmed the robustness and reproducibility of the final established population pharmacokinetic model of free methotrexate solution and methotrexate-loaded nanoformulations.

Here, the formulation refers to the difference between a free drug and a nanoformulated drug containing both nanoparticles and nanoemulsions.

### 3.3. Comparison of Pharmacokinetic Results between Methotrexate-Loaded Nanoparticles and Nanoemulsions

Although the zeta potential values of methotrexate-loaded nanoparticles and nanoemulsions in previous studies were slightly different, both formulations had negative charges. In addition, the particle sizes of the two formulations were almost the same, without any significant differences. The appearance of the formulations was also spherically identical, as confirmed by scanning electron microscopy (SEM) and transmission electron microscopy (TEM). The drug encapsulation efficiency values of both formulations were high, over 90%. Table 5 shows the comparison of basic physicochemical values for methotrexate-loaded nanoparticles and nanoemulsions prepared in previous studies [6,7]. However, as previously reported [17], nanoemulsions and nanoparticles can be classified into soft- and hard-type nanoformulations depending on the external flexibility of the formulation. Therefore, in this study, we attempted to confirm the formulation-dependent pharmacokinetic properties of soft- and hard-type formulations by comparing the pharmacokinetics of nanoemulsions and nanoparticles with similar particle sizes and shapes. 

**Figure 4:**
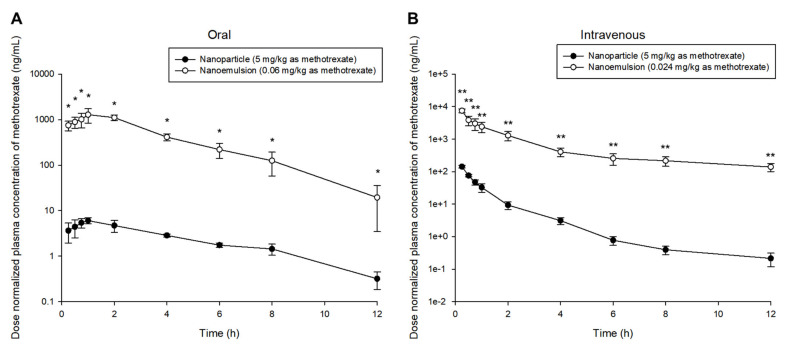
Dose-normalized mean plasma concentration-time profiles of methotrexate after oral (**A**) or intravenous (**B**) administration of methotrexate-loaded nanoparticles (-●-, 5 mg/kg as methotrexate) and methotrexate-loaded nanoemulsions (-○-, 0.06 or 0.024 mg/kg as methotrexate) in rats. Vertical bars represent standard deviation of the mean (*n* = 5). * *p* < 0.05 compared with the oral administration of nanoparticle. ** *p* < 0.05 compared with the intravenous administration of nanoparticle.

When comparing the in vivo pharmacokinetic profiles of methotrexate-loaded nanoemulsions and nanoparticles, significant differences were found between the two formulations. The plasma concentration of methotrexate was significantly higher following oral administration of nanoemulsions than nanoparticles (even though the administered dose of nanoemulsions was much smaller than that of nanoparticles). Figure S1 shows a comparison of the pharmacokinetic profiles of methotrexate-loaded nanoparticles and nanoemulsions following oral or intravenous administration. Considering that the oral dose of drug in nanoemulsions is about 83.33 times lower than that in nanoparticles, the higher plasma concentration in nanoemulsions translated to greater bioavailability of methotrexate. According to the dose-normalized plasma concentration-time profile (with the assumption of simple pharmacokinetic linearity), the concentration of methotrexate was significantly higher in the nanoemulsions than in the nanoparticles at all sampling time points. Figure 4 shows a comparison of the dose-normalized pharmacokinetic profile of methotrexate-loaded nanoparticles and nanoemulsions for oral or intravenous administration. These comparative results imply that the absorption of nanoemulsions into the systemic circulation through the gastrointestinal tract may be greater than that of nanoparticles when administered orally. This is probably because a nanoemulsion is a lipid-based formulation that can easily be absorbed into the systemic circulation in a process similar to the absorption of fat from the gastrointestinal tract. On the other hand, nanoparticles can enter the systemic circulation by absorption through Peyer’s patches, which have limited distribution in the gastrointestinal tract, or passive diffusion through some limited routes. Therefore, the oral bioavailability of nanoparticles may be lower than that of nanoemulsions due to the relatively low absorption of the former. In addition, nanoemulsions and nanoparticles can be classified as soft- and hard-type nanoformulations, respectively, according to their flexibility (solidity). Therefore, the oral bioavailability of nanoemulsions is higher than that of nanoparticles because nanoemulsions, which have relatively flexible surfaces, are freer to diffuse and move through the intracellular space than nanoparticles. The plasma concentration of methotrexate in nanoemulsions was significantly lower than that in nanoparticles until 1 or 4 h after intravenous administration of the drug-loaded nanoformulations (shown in Figure S1). However, reflecting the fact that the dose of methotrexate in nanoemulsions administered intravenously was about 208.33 times lower than that in the nanoparticles, the plasma concentration of methotrexate after administration of the nanoemulsions was considerably higher. In addition, according to the dose-normalized plasma concentration-time profiles, the concentration of methotrexate was significantly higher in the nanoemulsions than in the nanoparticles at all sampling time points (shown in Figure 4), i.e., assuming the pharmacokinetic linearity of methotrexate-loaded nanoformulations, the plasma concentration of methotrexate in nanoemulsions was significantly greater than the dose ratio with the nanoparticles. Moreover, the plasma concentration of methotrexate at 6–12 h after administration was significantly higher in nanoemulsions than in nanoparticles (Figures S1 and S4). Therefore, methotrexate-loaded nanoemulsions tend to stay longer in the blood than nanoparticles. The results of intravenous administration also suggest higher exposure and better pharmacokinetic properties of nanoemulsions than nanoparticles, which is similar to the results of oral administration.

In principle, the values of pharmacokinetic parameters such as T_1/2_, V, CL, etc. of pharmaceuticals consisting of small-molecule chemicals do not change with the route of administration or dosage in a linear system, and should not show significant differences between formulations. Nonetheless, there were significant differences between the pharmacokinetic parameter values (by non-compartment analysis) of methotrexate-loaded nanoemulsions and nanoparticles. These significant differences are thought to be caused by the differences in the pharmacokinetics of small-molecule chemicals and those enclosed in nanoformulations (as described above); also, the nanoformulations have different distribution properties in vivo depending on their size and surface characteristics. These characteristics are evident in non-biological complex drugs [20]. The pharmacokinetic parameters of the methotrexate-loaded nanoparticles and nanoemulsions are presented in Table 6. Following oral administration of methotrexate-loaded nanoemulsions and nanoparticles, significant differences were observed between the two formulations in all parameter values except T_max_. The dose-normalized AUC and C_max_ (AUC/Dose and C_max_/Dose) of methotrexate were 163–218 times higher in nanoemulsions than nanoparticles. The F value was also 1.89 times higher in methotrexate-loaded nanoemulsions than nanoparticles. This suggests that nanoemulsions tend to be relatively quickly absorbed (in large amounts) due to fat absorption when administered orally, but nanoparticles tend to be slowly absorbed due to slow dissolution and/or membrane restrictions that prevent particle uptake. Following intravenous administration, there were significant differences between nanoemulsions and nanoparticles in all parameters except T_max_, mirroring the results of oral administration. After oral administration, the T_1/2_ and MRT of nanoemulsions were reduced by 39% (2.59 to 1.58 h) and 30% (4.27 to 2.99 h), respectively, compared to nanoparticles. On the other hand, when administered intravenously, the T_1/2_ and MRT of nanoemulsions increased by about 394% (1.62 to 6.38 h) and 453% (0.91 to 4.12 h) compared to nanoparticles. The values of CL and V_ss_ significantly decreased to 98.8% (6984.15 to 80.94 mL/h/kg) and 94.5% (6301.14 to 338.68 mL/kg), respectively. This is thought to be because, following oral administration of methotrexate-loaded nanoemulsions, the properties of the nanoemulsions could be lost during absorption, in which case free methotrexate will end up in the blood; thus, the pharmacokinetics will reflect those of methotrexate itself. On the other hand, when administered intravenously, the characteristics of the nanoemulsions could be maintained. In addition, nanoparticles are structurally stronger than nanoemulsions in vivo and are more quickly taken up by the reticuloendothelial system (RES), which results in a significantly greater V than that of nanoemulsions. Moreover, the strength of the particles makes it easier for nanoparticles to be opsonized in blood and excreted through the kidneys more quickly than nanoemulsions [21,22], resulting in significantly higher values of CL and significantly lower values of T_1/2_.

**Figure 5:**
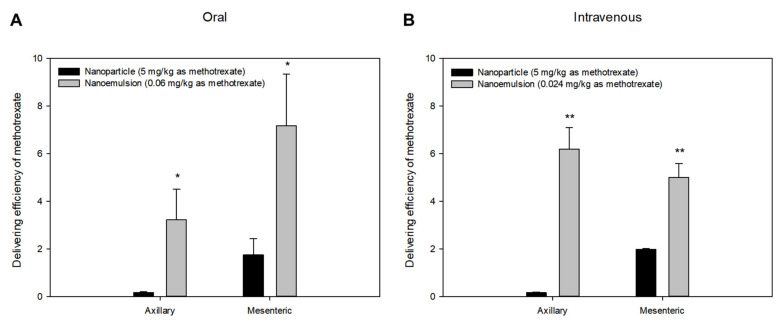
Mean delivering efficiencies of methotrexate to axillary and mesenteric lymph nodes at 2.5 h after oral (**A**) or intravenous (**B**) administration of methotrexate-loaded nanoparticles (-●-, 5 mg/kg as methotrexate) and methotrexate-loaded nanoemulsions (-○-, 0.06 or 0.024 mg/kg as methotrexate) in rats. Vertical bars represent standard deviation of the mean (*n* = 5). Delivering efficiency in each tissue was calculated as the ratio of the concentration in the tissues to the concentration in plasma. * *p* < 0.05 compared with the oral administration of nanoparticle. ** *p* < 0.05 compared with the intravenous administration of nanoparticle.

When the delivering efficiency (ratio of the concentration in tissues to the concentration in plasma) after oral or intravenous administration of methotrexate-loaded nanoemulsions and nanoparticles was calculated, significant differences between the two formulations were observed in axillary and mesenteric lymph nodes. Figure 5 shows the comparison of delivering efficiency in axillary and mesenteric lymph nodes according to oral or intravenous administration of methotrexate-loaded nanoparticles and nanoemulsions. In both axillary and mesenteric lymph nodes, the delivering efficiencies of methotrexate in nanoemulsions were significantly higher than those of methotrexate in nanoparticles. These results suggest that methotrexate-loaded nanoemulsions have a better capacity to deliver drug to lymphatic tissues than nanoparticles. The higher delivering efficiency to lymphatic tissues of nanoemulsions may be related to both the contribution of the fat absorption process (especially in the case of oral administration) and the easier accessibility to tight cell membranes due to better physical flexibility (especially in the case of intravenous administration).

As a result, the amount of drug delivered to the lymphatic tissues of nanoparticles may be less than that of nanoemulsions. Thus, following oral administration, the delivering efficiencies to mesenteric lymph nodes of both nanoparticles and nanoemulsions were significantly higher than those to axillary lymph nodes, and in the case of intravenous administration, the inverse was true, as shown in Figure 5. Moreover, this trend appeared much stronger in nanoemulsions due to the relatively strong contribution of fat absorption.

**Figure 6:**
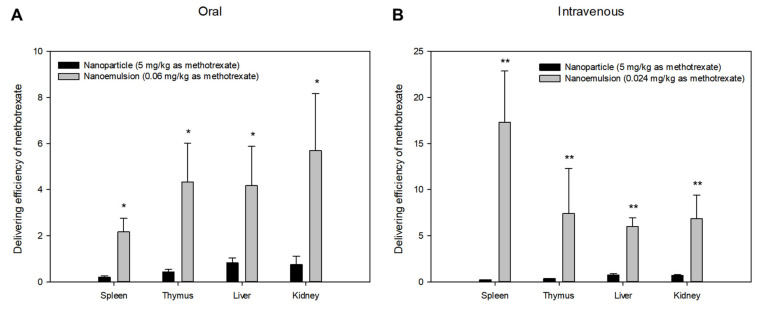
Mean delivering efficiencies of methotrexate to spleen, thymus, liver, and kidney at 2.5 h after oral (**A**) or intravenous (**B**) administration of methotrexate-loaded nanoparticles (-●-, 5 mg/kg as methotrexate) and methotrexate-loaded nanoemulsions (-○-, 0.06 or 0.024 mg/kg as methotrexate) in rats. Vertical bars represent standard deviation of the mean (*n* = 5). Delivering efficiency in each tissue was calculated as the ratio of the concentration in the tissues to the concentration in plasma. * *p* < 0.05 compared with the oral administration of nanoparticle. ** *p* < 0.05 compared with the intravenous administration of nanoparticle.

As in lymph nodes, the delivering efficiencies to spleen, thymus, liver and kidney after oral or intravenous administration of methotrexate-loaded nanoemulsions and nanoparticles were significantly different between the two formulations. Figure 6 shows the comparison of delivering efficiency in these tissues according to oral or intravenous administration. The drug delivering efficiency values of nanoemulsions to spleen, thymus, liver, and kidney were significantly higher than those of nanoparticles. The differences in delivering efficiency between formulations in these tissues may have similar causes as the differences seen in lymphatic tissues. The difference may also be because nanoemulsions, which are soft-type nanoformulations, can pass more easily between relatively tight cell membranes due to their superior physical flexibility, making delivery to various tissues easier. Considering that the V and V_ss_ values of nanoparticles are larger than those of nanoemulsions, many nanoparticles may be distributed to tissues other than the currently sampled tissues, i.e., in some of the currently sampled tissues, nanoemulsions show higher delivery efficiencies than nanoparticles, but the distribution of nanoparticles may be more important in other tissues such as fat, muscle, and so on.

### 3.4. Population Pharmacokinetic Modeling Approach to Comparing Methotrexate-Loaded Nanoparticles and Nanoemulsions

**Figure 7:**
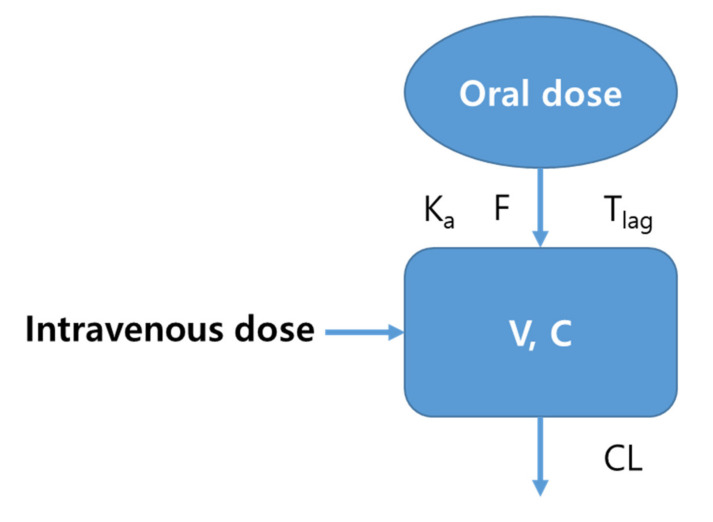
Structure of the population pharmacokinetic model of methotrexate-loaded nanoformulations (including nanoemulsions and nanoparticles).

A population pharmacokinetic modeling approach with NLME was used to investigate whether there is a pharmacokinetic difference between methotrexate-loaded nanoparticles and nanoemulsions, and if so, to what extent, and in what specific areas. All plasma concentration data for oral and intravenous administration of methotrexate-loaded nanoparticles and nanoemulsions were simultaneously fitted to the same model. The type of formulation (nanoparticles versus nanoemulsions) was set as the model’s covariate, and this covariate was evaluated for validity in the population pharmacokinetic model of methotrexate-loaded nanoformulations. When we applied the plasma concentration-time data of nanoparticles and nanoemulsions (according to oral or intravenous administration) to the base compartment models for each, the pharmacokinetics was best described by the 1-compartment model with first-order absorption with a lag-time, and first-order elimination. Figure 7 presents the structure of a related population pharmacokinetic model of methotrexate-loaded nanoformulations. 

We also attempted to use 2- or 3-compartment models, but these could not be applied to multiple subjects. Additionally, the absorption/elimination rates were better described as first-order than zero-order. The parameters, CL, V, K_a_, T_lag_, and F, were applied in this model. The residual error was best described using a log-additive error model. IIV of CL, V, K_a_, T_lag_, and F was incorporated into the base structural model. Although the total number of estimated parameters decreased by one when excluding IIV for each of the four parameters, this change caused a significant increase in the AIC and -2LL values without significant model improvement. These results were the same as the result of the comparison between free methotrexate solution and methotrexate-loaded nanoformulations. Table 7 summarizes the steps that were taken to develop the base structural model to represent methotrexate-loaded nanoparticles and nanoemulsions. The effect of covariates on the pharmacokinetic parameters of methotrexate-loaded nanoparticles and nanoemulsions was evaluated by sequentially applying the covariates to the base structural model established in the previous step. The covariate here was the difference in nanoformulation (methotrexate-loaded nanoparticles versus nanoemulsions). This covariate was reflected as categorical data. The fit of the model was judged by the change in OFV compared to the base model of the methotrexate-loaded nanoformulation (without covariates). When the difference in nanoformulation was considered to be a covariate of T_lag_, the OFV value did not decrease significantly with the increase in the number of parameters to be estimated. This implies that there was no significant difference in T_lag_ between formulations in this model-based approach. Including nanoformulation type in the base model as a covariate for V, CL, K_a_, and F (forward addition) resulted in an OFV that was lower than the significance threshold of 3.84 (*p* < 0.05). In particular, the decrease in OFV was greatest when nanoformulation type was considered to be a covariate of V. In this case, OFV was lower than 6.63 (*p* < 0.01), which was the criterion for model fit by backward elimination. Therefore, the data imply that use of nanoformulation type as a covariate for V is suitable and valid. In addition to V, the model fit by forward addition and backward elimination was lower than the reference values (3.84 or 6.63) despite the increase in the number of parameters to be estimated when the nanoformulation type was reflected as a covariate for CL, K_a_, and F. Therefore, the nanoformulation type was reflected in the four parameters, V, CL, K_a_, and F, as an effective covariate of the final population pharmacokinetic model for methotrexate-loaded nanoparticles and nanoemulsions. These results mirrored those of the comparison between free methotrexate solution and methotrexate-loaded nanoformulations. This means that all the methotrexate formulations (free methotrexate solution, methotrexate-loaded nanoparticles and nanoemulsions) could be included in the same population. Table 8 summarizes the covariate selection process for the final population pharmacokinetic model of methotrexate-loaded nanoparticles and nanoemulsions according to OFV. The final equation of the population pharmacokinetic model for the methotrexate-loaded nanoparticles and nanoemulsions was as follows:

V = tvV · (1 + dVdFormulation · formulation type _Nanoparticles = 0; Nanoemulsions = 1_) · exp(*ŋ*_V_) (6)

CL = tvCL · (1 + dCLdFormulation · formulation type _Nanoparticles = 0; Nanoemulsions = 1_) · exp(*ŋ*_CL_) (7)

T_lag_ = tvT_lag_ · exp(*ŋ*_Tlag_) (8)

K_a_ = tvK_a_ · (1 + dK_a_dFormulation · formulation type _Nanoparticles = 0; Nanoemulsions = 1_) · exp(*ŋ*_Ka_) (9)

F = tvF · (1 + dFdFormulation · formulation type _Nanoparticles = 0; Nanoemulsions = 1_) · exp(*ŋ*_F_) (10)

Equations (6)–(10) mean the equations for V, CL, T_lag_, K_a_, and F in the final population pharmacokinetic model (for the methotrexate-loaded nanoparticles and nanoemulsions), respectively. In the case of nanoparticles, the value of formulation type is 0, and in the case of nanoemulsions, the value of formulation type is 1.

Here, the formulation refers to the difference between methotrexate-loaded nanoparticles and nanoemulsions.

The parameter values estimated by the population pharmacokinetic model of methotrexate-loaded nanoparticles and nanoemulsions are presented in Table 9. The RSE (%) values of all parameters estimated in the final model were very low, 0.000–3.281%. The Eta shrinkage (%) values of V, CL, T_lag_, K_a_, and F were 0.085–0.392%, which were acceptable. The IIVs (%) of V, CL, K_a_, and F were as low as 0.238, 9.199, 0.002 and 3.206%, respectively, which were lower than the IIV values of the previously established base model for methotrexate-loaded nanoparticles and nanoemulsions (0.258, 13.027, 0.002, and 13.951%, respectively). This indirectly implies that in the final population pharmacokinetics model of methotrexate-loaded nanoparticles and nanoemulsions, it was appropriate to consider nanoformulation type as a covariate for V, CL, K_a_, and F. The correlations between V, CL, K_a_, and F and nanoformulation type were −0.986, −0.990, 1.552, and 0.193, respectively. This was similar to the values in the previous comparison between methotrexate-loaded nanoparticles and nanoemulsions (shown in Table 6), i.e., in the final model, the correlations between K_a_ and F and nanoformulation type are positive, and the correlations between V and CL and nanoformulation type are negative. This was consistent with the results of non-compartmental analysis, which showed that F, C_max_, etc. were higher in methotrexate-loaded nanoemulsions than methotrexate-loaded nanoparticles, whereas V and CL were lower. As a result, the covariate search results indicating that F increased and that V and CL were significantly decreased in nanoemulsions compared to nanoparticles according to the difference in nanoformulation type were well-reflected in the final population pharmacokinetics model of methotrexate-loaded nanoparticles and nanoemulsions. Given the results of population pharmacokinetic modeling for methotrexate-loaded nanoparticles and nanoemulsions, we can conclude that soft-type nanoformulations (such as nanoemulsions) have lower V and CL and larger K_a_ and F values than hard-type nanoformulations (such as nanoparticles). As the development of various soft- and hard-type nanoformulations and pharmacokinetic comparisons continues through a population pharmacokinetic model approach, the degree of pharmacokinetic difference between these two formulations and their correlations with parameters will be further elucidated. For the same reasons as discussed in Section 3.2, the estimates of T_lag_ and Eta for V, T_lag_, and K_a_ were nearly 0, as in the population pharmacokinetic model for free methotrexate solution and nanoformulations.

Here, the formulation refers to the difference between methotrexate-loaded nanoparticles and nanoemulsions.

### 3.5. Evaluation of the Population Pharmacokinetic Model for Comparing Methotrexate-Loaded Nanoparticles and Nanoemulsions

**Figure 8:**
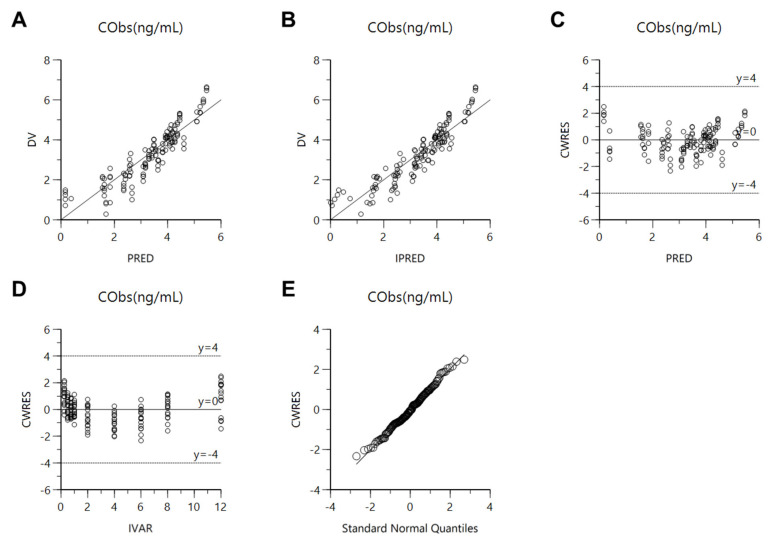
Goodness-of-fit plots of the final population pharmacokinetic model for methotrexate-loaded nanoformulations. (**A**) Population-predicted concentrations (PRED) vs. observed plasma concentration (DV); (**B**) Individual-predicted concentrations (IPRED) vs. DV; (**C**) PRED vs. CWRES; (**D**) Time (IVAR) vs. CWRES; (**E**) Quantile–quantile plot of the components of CWRES.

**Figure 9:**
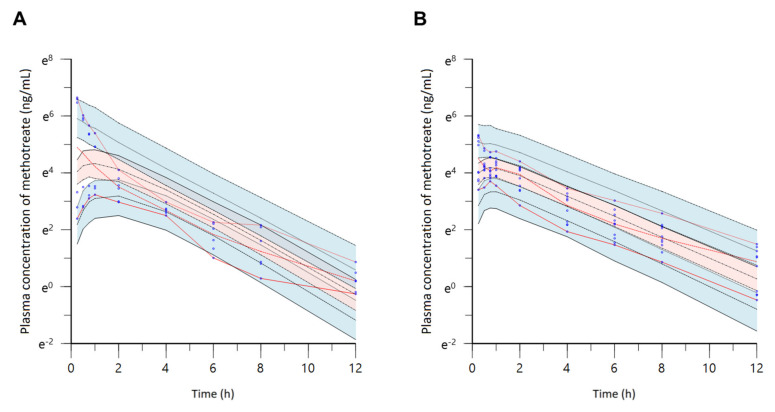
Visual predictive check of the final model for methotrexate-loaded nanoparticles (**A**) and nanoemulsions (**B**). Observed concentrations are depicted by dots. Black dashed lines indicate the 95th, 50th, and 5th percentiles of predicted concentrations. Blue shaded regions (with black boundary lines) indicate 95% confidence intervals for the predicted 5th and 95th percentiles. Red shaded regions indicate 95% confidence intervals for the predicted 50th percentiles. Red lines indicate the 95th, 50th, and 5th percentiles of observed concentrations.

The established population pharmacokinetic model of methotrexate-loaded nanoparticles and nanoemulsions was comprehensively evaluated using goodness-of-fit plots, visual predictive check, bootstrapping, and normalized prediction distribution error. Figure 8 shows the goodness-of-fit plots for the final population pharmacokinetics model of methotrexate-loaded nanoparticles and nanoemulsions. The methotrexate concentrations predicted by the population pharmacokinetic model of methotrexate-loaded nanoparticles and nanoemulsions in the population or individual showed relatively good agreement with the experimentally obtained observations. The CWRES were well-distributed symmetrically with respect to zero, i.e., CWRES were distributed at random without any specific bias. Additionally, the CWRES values never deviated from ±4 at any point of predicted concentration or time in the population. The quantile–quantile plot of the components of CWRES was close to a straight line where the *x*- and *y*-axes were symmetrical. Consequently, the goodness-of-fit plot presented in Figure 8 suggests that the final established population pharmacokinetic model of methotrexate-loaded nanoparticles and nanoemulsions had no graphically significant problems. Nevertheless, the goodness-of-fit plot results presented in Figure 3 were not superior to those in Figure 8 due to the inclusion of data obtained from administration of free methotrexate solution. In other words, when the data from the oral or intravenous administration of free methotrexate solution as well as methotrexate-loaded nanoformulations were considered in the model, the variability in the model’s predictions and parameters increased. This may be because the plasma concentration values of methotrexate according to oral or intravenous administration of free methotrexate solution were up to 10 times lower than those of methotrexate-loaded nanoformulations, resulting in a large degree of variance in the overall data. This observation is also associated with the fact that the AIC and OFV values of the base and final models presented in Table 1 and Table 2 were greater than those presented in Table 7 and Table 8. Table 10 shows the bootstrapping results for the final established population pharmacokinetic model of methotrexate-loaded nanoparticles and nanoemulsions. All the parameter values estimated in the final model were within the 95% confidence interval of the bootstrap analysis (number of replicates 1000). Additionally, the estimated values of the model parameters were similar to the median estimated by the bootstrap. Thus, bootstrapping analysis confirmed the robustness and reproducibility of the final population pharmacokinetic model of methotrexate-loaded nanoparticles and nanoemulsions. Figure 9 shows the results of a visual predictive check for the final model. The visual predictive check was performed with stratification by formulation type (nanoparticles or nanoemulsions). Figure S2 shows the results of the visual predictive check of the final population pharmacokinetic model of methotrexate-loaded nanoformulations (an integral one). Most of the observed values of methotrexate were well-distributed within the 90% prediction interval (5–95%) of predicted values. The visual predictive check results suggest that the population pharmacokinetic model of methotrexate-loaded nanoformulations described the overall experimental data relatively well. Furthermore, according to the visual predictive check results, the plasma concentration of methotrexate was higher in nanoemulsions (with low CL) than in methotrexate-loaded nanoparticles; this agrees with the CL correlation results between formulations presented in Table 9.

Here, the formulation refers to the difference between methotrexate-loaded nanoparticles and nanoemulsions.

**Figure 10:**
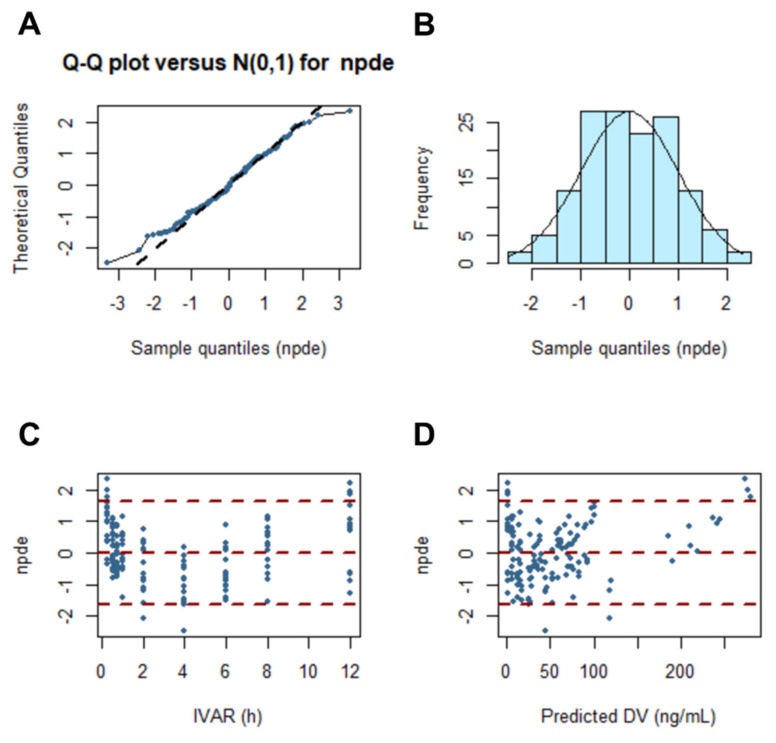
Normalized prediction distribution error for the final model of methotrexate-loaded nanoformulations. Quantile–quantile plots of normalized prediction distribution error vs. theoretical N (0, 1) distribution (**A**). Histogram showing the distribution of normalized prediction distribution error overlaid with density of the standard Gaussian distribution (**B**). Scatter plot of time vs. normalized prediction distribution error (**C**). Scatterplot of predictions vs. normalized prediction distribution error (**D**).

Figure 10 shows the results of normalized prediction distribution error analysis for the final population pharmacokinetic model of methotrexate-loaded nanoparticles and nanoemulsions. The normalized prediction distribution error considers the overall predicted distribution of each individual observation and processes multiple observations within a single subject, so it can be used to verify the normality of the data. The assumption that the differences between predictions and observations were normally distributed was acceptable. The quantile–quantile plots and histogram also confirmed the normality of the normalized prediction distribution error. Overall, the results of comprehensive evaluation of the final population pharmacokinetic model of methotrexate-loaded nanoparticles and nanoemulsions were all acceptable, and there were no major problems with the model.

### 3.6. Pharmacokinetic Comparison between Nanoformulations of Different Drugs

To determine whether the significant pharmacokinetic differences between methotrexate-loaded nanoparticles and nanoemulsions identified in this study are reflected in nanoformulation studies conducted in the past, a survey of the relevant literature was conducted [4,5,8,9,10,11,12,13,14,15,16]. Table 11 summarizes the pharmacokinetic findings of this literature search [4,5,8,9,10,11,12,13,14,15,16]. In many previous nanoformulation studies, each formulation that was examined had very different physicochemical properties. In particular, the sizes of nanoformulations varied greatly. Therefore, a direct comparison between the pharmacokinetics of different nanoformulations such as that in our current study was virtually impossible. This is because, above all else, the pharmacokinetic properties of nanoformulations are primarily influenced by size. The comparison of the pharmacokinetics of methotrexate-loaded nanoparticles and nanoemulsions [6,7] done in this study was novel and is expected to provide a useful reference for nanoformulations of various drugs in the future, i.e., the data that confirms the difference in pharmacokinetics between nanoparticles and nanoemulsions, which serve as representative hard- and soft-type nanoformulations, is useful evidence for nanoformulation selection and the creation of strategies to improve the bioavailability and delivery efficiency of specific drugs.

## 4. Conclusions

In this study, the pharmacokinetic differences between nanoparticles and nanoemulsions, which correspond to hard- and soft-type nanoformulations, were analyzed and compared. In addition, the pharmacokinetic differences between free methotrexate solution as a control group and methotrexate-loaded nanoformulations as the experimental groups were compared through population pharmacokinetic model analysis using NLME. Comparing pharmacokinetics between formulations using population pharmacokinetic models is a new approach that has not been previously reported, and it is thought that it will serve as a very useful tool in future comparative studies between nanoformulations. According to the pharmacokinetic comparison between methotrexate formulations, methotrexate-loaded nanoformulations had significantly higher F and lower CL values compared to free methotrexate solution; this finding was confirmed by both population pharmacokinetic model analysis and non-compartmental analysis. In addition, the higher F and lower CL values in nanoemulsions than methotrexate-loaded nanoparticles were confirmed by the same approaches that were used to compare free methotrexate solution and methotrexate-loaded nanoformulations. To further supplement and clarify the results of this study, there is a need to conduct an additional comparative study of pharmacokinetics between nanoformulations with no significant difference in physical properties.

## Figures and Tables

**Table 1:** Steps used to build the base structural model for free methotrexate solution and methotrexate-loaded nanoformulations.

Model	Description	*n*-Parameter	−2LL	AIC	Δ − 2LL	ΔAIC	Compared with	Residual Error Model	Compartment Model
**Absorption model**									
01	No T_lag_	9	3255.48	3280.13			-	additive error model	1-compartment
02 *	Add T_lag_	11	2442.04	2464.04	−813.44	−816.08	01	additive error model	1-compartment
**Residual error model**									
02	Additive	11	2442.04	2464.04	0.00	0.00	02	additive error model	1-compartment
02-01	Proportional	11	2192.37	2214.37	−249.67	−249.67	02	proportional error model	1-compartment
02-02	Power	11	2442.04	2464.04	0.00	0.00	02	power error model	1-compartment
02-03	Mixed	12	2150.31	2174.31	−291.73	−289.73	02	mixed error model	1-compartment
02-04 *	Log-additive	11	759.13	781.13	−1682.91	−1682.91	02	log-additive error model	1-compartment
**IIV model**									
02-04-01	Remove IIV V	10	1060.56	1080.56	301.43	299.43	02-04	log-additive error model	1-compartment
02-04-02	Remove IIV CL	10	1154.02	1174.02	394.89	392.89	02-04	log-additive error model	1-compartment
02-04-03	Remove IIV K_a_	10	789.73	809.73	30.60	28.60	02-04	log-additive error model	1-compartment
02-04-04	Remove IIV T_lag_	10	1085.34	1105.34	326.21	324.21	02-04	log-additive error model	1-compartment
02-04-05	Remove IIV F	10	860.97	880.97	101.84	99.84	02-04	log-additive error model	1-compartment

* indicates the selected models at each step. IIV was set as the exponential error model.

**Table 2:** Stepwise search for covariates in the models of free methotrexate solution and methotrexate-loaded nanoformulations.

Model	Description	OFV	ΔOFV	Compared with	*n*-Parameter
02-04	Base model	759.128			11
02-04-C1	Formulation on V	754.284	−4.843	02-04	12
02-04-C2	Formulation on CL	752.102	−7.026	02-04	12
02-04-C3	Formulation on T_lag_	761.168	2.040	02-04	12
02-04-C4	Formulation on K_a_	754.534	−4.594	02-04	12
02-04-C5	Formulation on F	754.132	−4.995	02-04	12
02-04-C6	Formulation on CL & V	742.395	−9.707	02-04-C2	13
02-04-C7	Formulation on CL & K_a_	745.284	−6.818	02-04-C2	13
02-04-C8	Formulation on CL & F	744.399	−7.703	02-04-C2	13
02-04-C9	Formulation on CL & V & F	732.588	−9.807	02-04-C6	14
02-04-C10	Formulation on CL & V & K_a_	735.805	−6.590	02-04-C6	14
02-04-C11 *	Formulation on CL & V & F & K_a_	722.746	−9.842	02-04-C9	15

* indicates the final selected model.

**Table 3:** Estimated population pharmacokinetic parameters for free methotrexate solution and methotrexate-loaded nanoformulation in the final model.

Parameters	Units	Estimate	SE	RSE (%)	Shrinkage (%)	IIV (%)
tvV	L/kg	14.889	1.388	9.320	-	-
tvCL	L/h/kg	14.577	1.572	10.785	-	-
tvT_lag_	h	0.000	0.000	31.713	-	-
tvK_a_	1/h	0.582	0.147	25.291	-	-
tvF	-	0.272	0.030	11.055	-	-
dVdFormulation	-	0.429	0.136	31.591	-	-
dCLdFormulation	-	−0.355	0.086	24.316	-	-
dK_a_dFormulation	-	10.883	1.574	14.465	-	-
dFdFormulation	-	4.246	1.233	29.027	-	-
ω^2^_V_	-	0.000	0.000	7.332	0.393	0.252
ω^2^_CL_	-	0.338	0.153	45.227	0.173	58.130
ω^2^_Tlag_	-	0.000	0.000	0.014	0.600	0.052
ω^2^_Ka_	-	0.000	0.000	0.014	0.465	0.002
ω^2^_F_	-	0.386	0.121	31.254	0.316	62.161
σ	-	1.269	0.132	10.374	-	-

SE: standard error. RSE: relative standard error.

**Table 4:** Estimated population pharmacokinetic parameter values of free methotrexate solution and methotrexate-loaded nanoformulations, and bootstrap validation (*n* = 1000).

Parameters	Units	Final Model		Bootstrapping	
		Estimate	95% Confidence Interval	Median	95% Confidence Interval
tvV	L/kg	14.889	12.169–17.608	13.267	10.004–16.531
tvCL	L/h/kg	14.577	11.496–17.659	12.840	9.142–16.537
tvT_lag_	h	0.000	0.000–0.000	0.000	0.000–0.000
tvK_a_	1/h	0.582	0.293–0.870	0.554	0.207–0.900
tvF	-	0.272	0.213–0.331	0.239	0.168–0.309
dVdFormulation	-	0.429	0.164–0.695	0.407	0.088–0.726
dCLdFormulation	-	−0.355	−0.524–−0.186	−0.362	−0.565–−0.159
dK_a_dFormulation	-	10.883	7.797–13.968	9.999	6.296–13.701
dFdFormulation	-	4.246	1.830–6.662	3.910	1.011–6.809
ω^2^_V_	-	0.000	0.000–0.000	0.000	0.000–0.000
ω^2^_CL_	-	0.338	0.038–0.637	0.338	0.022–0.697
ω^2^_Tlag_	-	0.000	0.000–0.000	0.000	0.000–0.000
ω^2^_Ka_	-	0.000	0.000–0.000	0.000	0.000–0.000
ω^2^_F_	-	0.386	0.150–0.623	0.386	0.102–0.670
σ	-	1.269	1.011–1.527	1.262	0.952–1.572

**Table 5:** Comparison of the basic physicochemical properties of different methotrexate-loaded nanoformulations.

Properties	Methotrexate-Loaded PLGA Nanoparticles	Methotrexate-Loaded Nanoemulsions
Particle size	163.7 ± 10.25 nm	173.77 ± 5.76 nm
Zeta potential	−20.4 ± 1.54 mV	−35.63 ± 0.78 mV
Encapsulation efficiency	93.3 ± 0.5%	90.37 ± 0.96%
Shape	Spherical form	Spherical form
References	Jang et al. (2019) [6]	Jang et al. (2020) [7]

**Table 6:** Comparison of estimated pharmacokinetic parameters of methotrexate-loaded nanoformulations after a single dose to rats (*n* = 5) by non-compartmental analysis.

Parameters	Oral (5 mg/kg as Methotrexate)	Oral (0.06 mg/kg as Methotrexate)	Intravenous (5 mg/kg as Methotrexate)	Intravenous (0.024 mg/kg as Methotrexate)
	Nanoparticles	Nanoemulsions	Nanoparticles	Nanoemulsions
AUC_0-t_ (ng·h/mL)	142.05 ± 7.00	288.35 ± 51.14 *	720.15 ± 81.74	268.94 ± 41.85 **
AUC_0-∞_ (ng·h/mL)	148.44 ± 7.43	291.34 ± 54.01 *	722.53 ± 82.58	300.56 ± 36.10 **
C_max_ (ng/mL)	31.19 ± 5.15	81.72 ± 23.01 *	713.07 ± 62.83	180.05 ± 24.79 **
C_0_ (ng/mL)	-	-	1344.57 ± 200.87	366.10 ± 76.22 **
AUC_0-t_/Dose (h·kg/mL)	2.84 10^−5^ ± 1.40 10^−6^	4.81 10^−3^ ± 8.52 10^−4^ *	1.44 10^−4^ ± 1.64 10^−5^	1.12 10^−2^ ± 1.74 10^−3^ **
AUC_0-∞_/Dose (h·kg/mL)	2.97 10^−5^ ± 1.49 10^−6^	4.86 10^−3^ ± 9.00 10^−4^ *	1.45 10^−4^ ± 1.65 10^−5^	1.25 10^−2^ ± 1.50 10^−3^ **
C_max_/Dose (kg/mL)	6.24 10^−6^ ± 1.03 10^−6^	1.36 10^−3^ ± 3.83 10^−4^ *	1.43 10^−4^ ± 1.26 10^−5^	7.50 10^−3^ ± 1.03 10^−3^ **
T_max_ (h)	0.92 ± 0.14	1.35 ± 0.60	0.25 ± 0.00	0.25 ± 0.00
T_1/2_ (h)	2.59 ± 0.66	1.58 ± 0.30*	1.62 ± 0.61	6.38 ± 1.77 **
CL (mL/h/kg)	-	-	6984.15 ± 840.71	80.94 ± 11.38 **
V (mL/kg)	-	-	16146.33 ± 5631.17	748.38 ± 232.69 **
MRT (h)	4.27 ± 0.76	2.99 ± 0.37 *	0.91 ± 0.05	4.12 ± 1.56 **
V_ss_ (mL/kg)	-	-	6301.14 ± 404.35	338.68 ± 146.77 **
F (%)	20.54	38.77 *	-	-

C_0_ is the extrapolated drug plasma concentration at 0 h. MRT is the mean residence time of drug. V_ss_ is the predicted volume of drug distribution when a steady state was reached. F is the absolute bioavailability of the drug for each nanoformulation. * *p* < 0.05 compared with oral administration of nanoparticles. ** *p* < 0.05 compared with intravenous administration of nanoparticles.

**Table 7:** Steps used to build the base structural model for methotrexate-loaded nanoparticles and nanoemulsions.

Model	Description	*n*-Parameter	−2LL	AIC	Δ − 2LL	ΔAIC	Compared with	Residual Error Model	Compartment Model
**Absorption model**									
01	No T_lag_	9	1545.89	1563.89	-	-	-	additive error model	1-compartment
02 *	Add T_lag_	11	1505.72	1527.72	−40.17	−36.17	01	additive error model	1-compartment
**Residual error model**									
02	Additive	11	1505.72	1527.72	0.00	0.00	02	additive error model	1-compartment
02-01	Proportional	11	1412.20	1434.20	−93.52	−93.52	02	proportional error model	1-compartment
02-02	Power	11	1545.72	1567.72	40.00	40.00	02	power error model	1-compartment
02-03	Mixed	12	1545.72	1569.72	40.00	42.00	02	mixed error model	1-compartment
02-04 *	Log-additive	11	498.53	520.53	−1007.19	−1007.19	02	log-additive error model	1-compartment
**IIV model**									
02-04-01	Remove IIV V	10	595.61	615.61	97.08	95.08	02-04	log-additive error model	1-compartment
02-04-02	Remove IIV CL	10	595.60	615.60	97.07	95.07	02-04	log-additive error model	1-compartment
02-04-03	Remove IIV K_a_	10	595.61	615.61	97.08	95.08	02-04	log-additive error model	1-compartment
02-04-04	Remove IIV T_lag_	10	595.61	615.61	97.08	95.08	02-04	log-additive error model	1-compartment
02-04-05	Remove IIV F	10	534.28	554.28	35.75	33.75	02-04	log-additive error model	1-compartment

* indicates the models selected at each step. IIV was set as the exponential error model.

**Table 8:** Stepwise search for covariates in the models of methotrexate-loaded nanoparticles and nanoemulsions.

Model	Description	OFV	ΔOFV	Compared with	n-Parameter
02-04	Base model	498.535			11
02-04-C1	Formulation on V	338.410	−160.125	02-04	12
02-04-C2	Formulation on CL	492.153	−6.382	02-04	12
02-04-C3	Formulation on T_lag_	496.267	−2.267	02-04	12
02-04-C4	Formulation on K_a_	493.304	−5.231	02-04	12
02-04-C5	Formulation on F	465.927	−32.608	02-04	12
02-04-C6	Formulation on V & F	331.262	−7.148	02-04-C1	13
02-04-C7	Formulation on V & CL	273.319	−65.091	02-04-C1	13
02-04-C8	Formulation on V & K_a_	331.262	−7.148	02-04-C1	13
02-04-C9	Formulation on V & CL & K_a_	246.629	−26.690	02-04-C7	14
02-04-C10	Formulation on V & CL & F	259.065	−14.254	02-04-C7	14
02-04-C11 *	Formulation on V & CL & K_a_ & F	220.361	−26.268	02-04-C9	15

* indicates the final selected model.

**Table 9:** Estimated population pharmacokinetic parameters for methotrexate-loaded nanoformulations in the final model.

Parameters	Units	Estimate	SE	RSE (%)	Shrinkage (%)	IIV (%)
tvV	L/kg	18.832	0.065	0.348	-	-
tvCL	L/h/kg	9.167	0.252	2.739	-	-
tvT_lag_	h	0.000	0.000	0.172	-	-
tvK_a_	1/h	0.714	0.023	3.281	-	-
tvF	-	0.334	0.006	1.883	-	-
dVdFormulation	-	−0.986	0.001	0.097	-	-
dCLdFormulation	-	−0.990	0.001	0.054	-	-
dK_a_dFormulation	-	1.552	0.004	0.264	-	-
dFdFormulation	-	0.193	0.000	0.114	-	-
ω^2^_V_	-	0.000	0.000	0.000	0.201	0.238
ω^2^_CL_	-	0.008	0.000	1.317	0.085	9.199
ω^2^_Tlag_	-	0.000	0.000	0.000	0.392	0.052
ω^2^_Ka_	-	0.000	0.000	0.000	0.224	0.002
ω^2^_F_	-	0.001	0.000	0.042	0.116	3.206
σ	-	0.552	0.005	0.967	-	-

SE: standard error. RSE: relative standard error.

**Table 10:** Estimated population pharmacokinetic parameter values of methotrexate-loaded nanoformulations, and bootstrap validation (*n* = 1000).

Parameters	Units	Final Model		Bootstrapping	
		Estimate	95% Confidence Interval	Median	95% Confidence Interval
tvV	L/kg	18.832	18.827–18.835	18.832	18.826–18.836
tvCL	L/h/kg	9.167	9.166–9.175	9.168	9.165–9.176
tvT_lag_	h	0.000	0.000–0.000	0.000	0.000–0.000
tvK_a_	1/h	0.714	0.714–0.714	0.714	0.713–0.715
tvF	-	0.334	0.334–0.334	0.334	0.334–0.334
dVdFormulation	-	−0.986	−0.988–−0.984	−0.986	−0.988–−0.984
dCLdFormulation	-	−0.990	−0.991–−0.989	−0.990	−0.991–−0.989
dK_a_dFormulation	-	1.552	1.552–1.552	1.552	1.552–1.552
dFdFormulation	-	0.193	0.193–0.193	0.193	0.193–0.193
ω^2^_V_	-	0.000	0.000–0.000	0.000	0.000–0.000
ω^2^_CL_	-	0.008	0.008–0.009	0.008	0.007–0.009
ω^2^_Tlag_	-	0.000	0.000–0.000	0.000	0.000–0.000
ω^2^_Ka_	-	0.000	0.000–0.000	0.000	0.000–0.000
ω^2^_F_	-	0.001	0.001–0.001	0.001	0.001–0.001
σ	-	0.552	0.552–0.552	0.552	0.551–0.553

**Table 11:** Summary of pharmacokinetic data of previously reported nanoparticles and nanoemulsion formulations.

8	Formulation	Size and Zeta Potential	Subjects	Pharmacokinetic Parameters						References
				T_1/2_	T_max_	C_max_	AUC_0–∞_	CL/F	V_d_	
Docetaxel	PLA-PLGA nanoparticles	216 ± 1 nm −3.11 ± 0.28 mV	Mice (*n* = 3) 10 mg/kg intravenous	6.6–7.4 (h)	-^a^	19583–26753 (ng/mL)	82743–95692 (h·ng/mL; AUC_0~t_)	105–121 (mL/h/kg)	943–1278 (L/kg)	Chu et al. (2013) [14]
	PEG-PLGA nanoparticles	186.7 ± 2.9 nm −25.9 ± 3.5 mV	Mice (*n* = 4) 5 mg/kg intravenous	15.87 ± 1.66 (h)	-^a^	-^a^	9221 ± 4709 (h·ng/mL)	12.54 ± 4.53 (mL/h)	290.41 ± 116.32 (mL)	Rafiei et al. (2017) [8]
	PLGA nanoparticles	123.6 ± 9.5 nm −28.3 ± 1.2 mV	Mice (*n* = 4) 5 mg/kg intravenous	6.05±0.78 (h)	-^a^	-^a^	6601 ± 2,655 (h·μg/mL)	17.23 ± 7.16 (L/h)	150.81 ± 74.18 (L)	Rafiei et al. (2017) [8]
	Nanoemulsions	120–140 nm −48–−29 mV	Mice (*n* = 3) 10 mg/kg intravenous	6.1 ± 3.8 (h)	-^a^	3660 ± 433 (ng/mL)	2840 ± 55 (h·ng/L)	3.5 ± 0.1 (L/h/kg)	31 ± 19 (L/kg)	Patel et al. (2018) [4]
	SEDDS	167.3 ± 2.30	Rat (*n* = 6) 10 mg/kg oral	34.83 ± 7.70 (h)	0.17 (h)	125.5 ± 2.50 (ng/mL)	260.23 ± 51.8 (h·ng/mL)	28.31 ± 3.33 (L/h/kg)	1460.33 ± 484.28 (L/kg)	Valicherla et al. (2016) [9]
Tacrolimus	SEDDS	43.4 ± 3.58 nm −41.26 ± 1.94 mV	Rat (*n* = 6) 5 mg/kg oral	-^a^	2.3 ± 0.5 (h)	205.8 ± 32.8 (ng/mL)	1745.2 ± 132.3 (h·ng/mL)	-^a^	-^a^	Cho et al. (2015) [13]
	PLGA nanoparticles	218 ± 51 nm −28.2 ± 4.3 mV	Rat (*n* = 6) 1 mg/kg intravenous	3.157 ± 1.274 (h)	-^a^	-^a^	566.187 ± 235.008 (h·ng/mL)	10.29 ± 4.81 (mL/min)	-^a^	Shin et al. (2010) [16]
	PEG-PLGA nanoparticles	220 ± 33 nm −24.5 ± 5.7 mV	Rat (*n* = 6) 1 mg/kg intravenous	269.32 ± 136.16 (min)	-^a^	-^a^	39526.18 ± 3411.35 (min·ng/mL)	7.90 ± 0.62 (mL/min)	-^a^	Shin et al. (2010) [16]
Paclitaxel	SEDDS	18.4 ± 0.912 nm 12.5 ± 1.66 mV,	Rat (*n* = 5) 20 mg/kg oral	-^a^	1.7 ± 0.2 (h)	259.5 ± 7.5 (ng/mL)	3308.5 ± 486.2 (h·ng/mL)	-^a^	-^a^	Cho et al. (2016) [12]
	PEGylated nanoparticles	178–180 nm −40.3–−39.5 mV	Rat (*n* = 6) 10 mg/kg oral	6.2–9.3 (h)	3.0–5.8 (h)	1.9–2.1 (μg/mL)	32–56 (h·μg/mL)	-^a^	-^a^	Zabaleta et al. (2012) [15]
	PLGA nanoparticles	308.6 ± 6.22 nm −10.70 ± 0.21 mV	Rat (*n* = 3) 5 mg/kg intravenous	28.48 ± 0.99 (h)	-^a^	951.9 ± 47.5 (ng/mL)	2915.46 ± 145.54 (h·ng/mL)	0.80 ± 0.03 (L/h)	-^a^	Mandal et al. (2018) [5]
5-FU	Nanoemulsions	20.3 ± 0.22 nm −4.65 ± 1.68 mV	Rat (*n* = 4) 20 mg/kg oral	1.386 ± 0.146 (h)	0.833 ± 0.289 (h)	0.164 ± 0.044 (μg/mL)	0.360 ± 0.091 (h·μg/mL)	-^a^	-^a^	Pangeni et al. (2016) [10]
	PLA nanoparticles	294 ± 5 nm	Rat (*n* = 5) 50 mg/kg oral	3.46 ± 0.14 (h)	6 (h)	467.34 ± 0.75 (ng/mL)	2200.53 ± 1.82 (h·ng/mL)	2.4 ± 0.03 × 10^4^ (L/h/kg)	12.0 ± 0.02 × 10^4^ (L/kg)	De Mattos et al. (2016) [11]
	PLA-PEG nanoparticles	283 ± 10 nm	Rat (*n* = 5) 50 mg/kg oral	3.01 ± 0.19 (h)	6 (h)	487.34 ± 1.79 (ng/mL)	2281.1 ± 2.08 (h·ng/mL)	2.4 ± 0.02 × 10^4^ (L/h/kg)	10.7 ± 0.02 × 10^4^ (L/kg)	De Mattos et al. (2016) [11]

^a^ indicates that specific values are not clearly presented in the references.

## Data Availability

The data presented in this study are available in the article and Supplementary Materials.

## Supplementary Materials

The following are available online at https://www.mdpi.com/article/10.3390/pharmaceutics13071050/s1, Figure S1. Mean plasma concentration-time profiles of methotrexate after oral (A) or intravenous (B) administration of methotrexate-loaded nanoparticles (-●-, 5 mg/kg as methotrexate) and methotrexate-loaded nanoemulsions (-○-, 0.06 or 0.024 mg/kg as methotrexate) in rats. Vertical bars represent standard deviation of the mean (*n* = 5); Figure S2. Visual predictive check of the final model for methotrexate-loaded nanoformulations (including nanoparticles and nanoemulsions). Observed concentrations are depicted by dots. Black dashed lines indicate the 95th, 50th, and 5th percentiles of predicted concentrations. Blue shaded regions (with black boundary lines) indicate 95% confidence intervals for the predicted 5th and 95th percentiles. Red shaded regions indicate 95% confidence intervals for the predicted 50th percentiles. Red lines indicate the 95th, 50th, and 5th percentiles of observed concentrations.

## Abbreviations

UHPLC-ESI-MS/MS	Ultrahigh-performance liquid chromatography-electrospray ionization-mass spectrometry
SD	Standard deviation
NLME	Nonlinear mixed effects model
AIC	Akaike information criterion
SC	Schwarz criterion
WRSS	Weighted residual sum of squares
IIV	Interindividual variability
OFV	Objective function value
SE	Standard error
RSE	Relative standard error
MRT	Mean residence time
AUMC	Area under the first moment curve
SEM	Scanning electron microscopy
TEM	Transmission electron microscopy
RES	Reticuloendothelial system
