# Supplementary material for: Pharmacokinetic Comparison between Methotrexate-Loaded Nanoparticles and Nanoemulsions as Hard- and Soft-Type Nanoformulations: A Population Pharmacokinetic Modeling Approach
Source: Pharmaceutics. 2021 Jul 9;13(7):1050. doi: 10.3390/pharmaceutics13071050 (PMC8309067; doi:10.3390/pharmaceutics13071050)
Supplement: Supplementary file 1 [file pharmaceutics-13-01050-s001.zip › pharmaceutics-1261853-supplementary.pdf]

pharmaceutics

MDPI

Supplementary Materials: Pharmacokinetic Comparison Between Methotrexate-Loaded Nanoparticles and Nanoemulsions as Hard- and Soft-Type Nanoformulations: A Population Pharmacokinetic Modeling Approach

Seung-Hyun Jeong, Ji-Hun Jang and Yong-Bok Lee

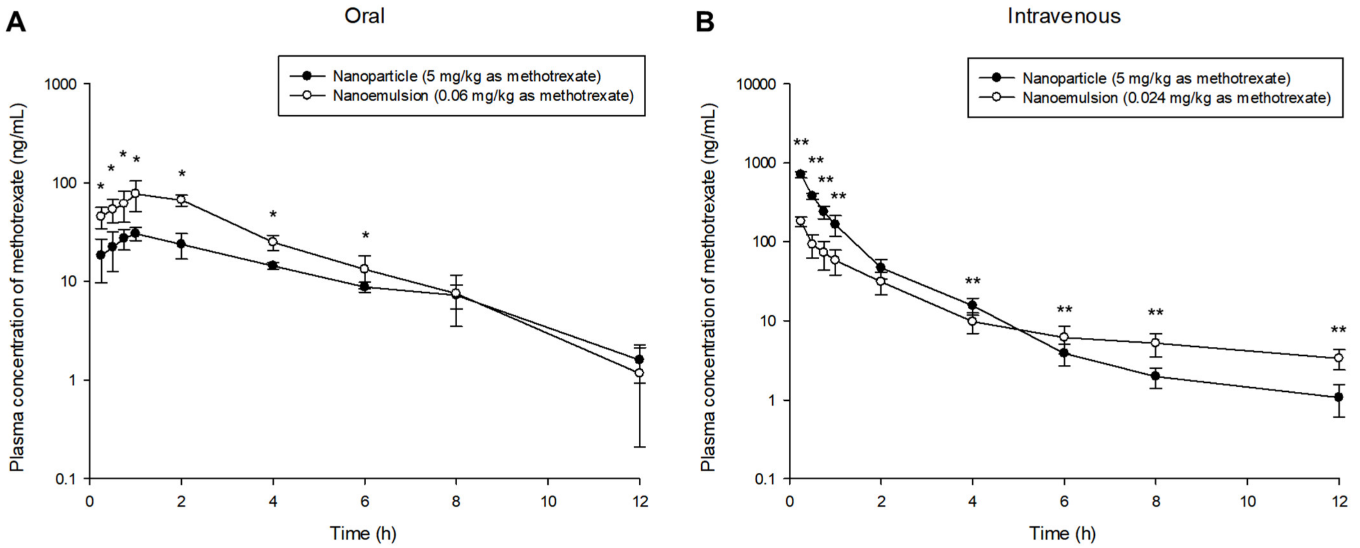

Figure S1. Mean plasma concentration-time profiles of methotrexate after oral (A) or intravenous (B) administration of methotrexate-loaded nanoparticles (-●-, 5 mg/kg as methotrexate) and methotrexate-loaded nanoemulsions (-○-, 0.06 or 0.024 mg/kg as methotrexate) in rats. Vertical bars represent standard deviation of the mean ($n = 5$). * $p < 0.05$ compared with the oral administration of nanoparticle. ** $p < 0.05$ compared with the intravenous administration of nanoparticle.

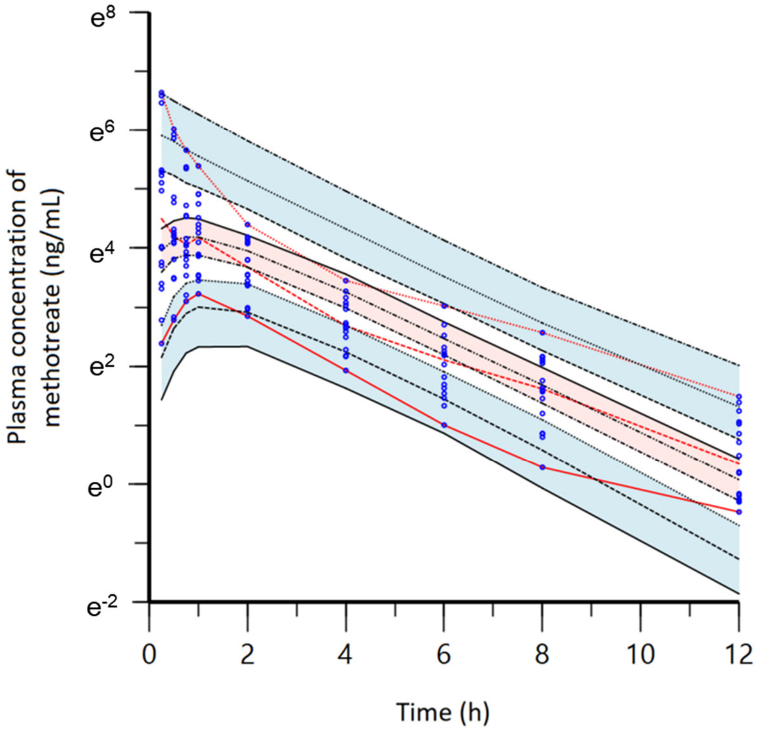

Figure S2. Visual predictive check of the final model for methotrexate-loaded nanoformulations (including nanoparticles and nanoemulsions). Observed concentrations are depicted by dots. Black dashed lines indicate the 95th, 50th, and 5th percentiles of predicted concentrations. Blue shaded regions (with black boundary lines) indicate 95% confidence intervals for the predicted 5th and 95th percentiles. Red shaded regions indicate 95% confidence intervals for the predicted 50th percentiles. Red lines indicate the 95th, 50th, and 5th percentiles of observed concentrations.
